# A diabetic patient increased premature ventricular contractions after using liraglutide: a case report

**DOI:** 10.3389/fcvm.2024.1332754

**Published:** 2024-01-31

**Authors:** Lilan Huang, He Yu, Ying Fang

**Affiliations:** ^1^ Medical School of the Ministry of Medicine, Wuhan University of Science and Technology, Wuhan, China; ^2^ Department of Endocrinology, CR & WISCO General Hospital Affiliated to Wuhan University of Science and Technology, Wuhan, China

**Keywords:** glucagon-like peptide-1 receptor agonists (GLP-1RAs), premature ventricular contractions, liraglutide, diabetes mellitus, arrhythmia

## Abstract

The common adverse reactions of liraglutide are hypoglycemia and gastrointestinal reactions. This case reports a patient with type 2 diabetes mellitus who had an increase in ventricular premature beats after using liraglutide and a decrease in ventricular premature beats after stopping liraglutide, suggesting that clinicians should rule out the possibility of drug-related adverse reactions when using liraglutide in the treatment of diabetes mellitus complicated with coronary heart disease and arrhythmia.

## 1 Introduction

Glucagon-like peptide-1 receptor agonists (GLP-1RAs) are relatively novel anti-hyperglycemic drugs that mimic the effects of endogenous GLP-1 and contribute to improved glycemic control and weight loss. Evidence has accumulated that GLP-1RAs could also be associated with improved cardiovascular outcomes and a reduction in major adverse cardiovascular events (MACEs) (1). Recent findings have shown that liraglutide significantly reduced the combined primary outcome of major adverse cardiovascular events in large cardiovascular outcome trials, elevating the importance of understanding how activation of the GLP-1R translates into clinical cardiovascular benefit (2). Liraglutide is a novel GLP-1 analogue that is now widely recommended for the treatment of type 2 diabetes in combination with cardiovascular diseases (3). The most common adverse effects of liraglutide are gastrointestinal symptoms, including nausea, diarrhea, and vomiting. Currently, the relationship between GLP-1RAs and arrhythmias requires further explored.

## 2 Case presentation

**Figure 1:**
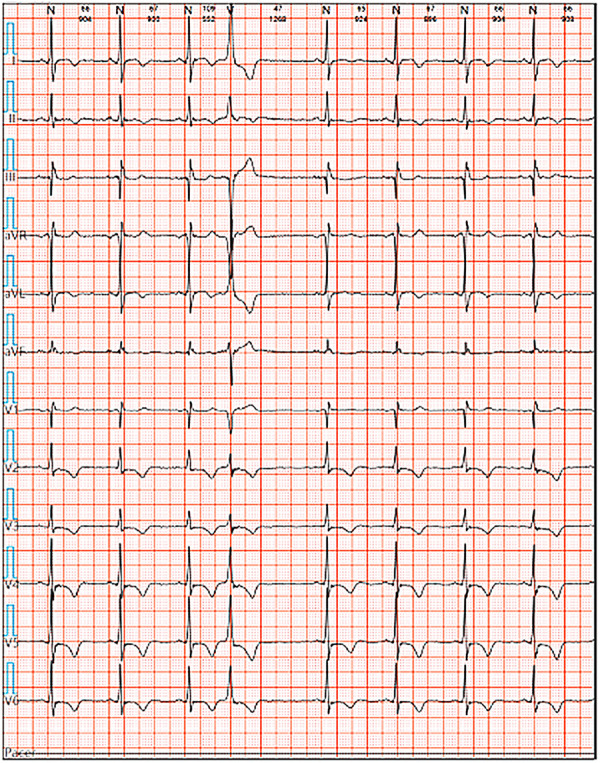
Ambulatory ECG before liraglutide injection on Feb 09.

**Figure 2:**
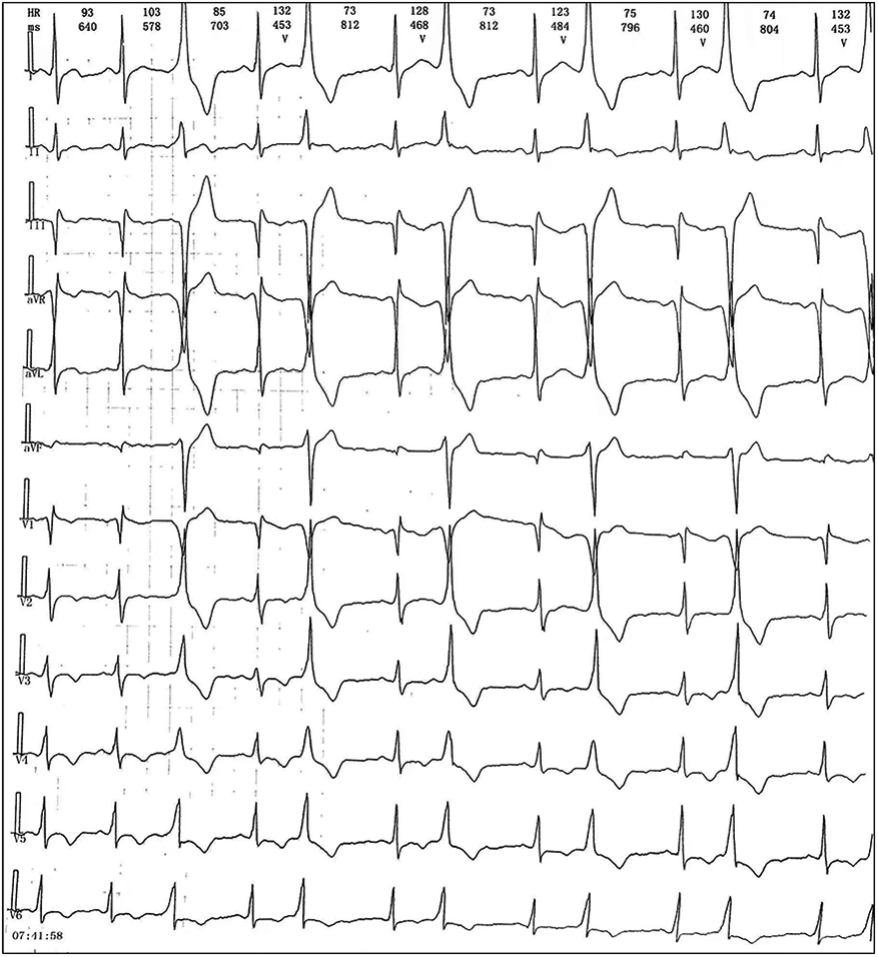
Ambulatory ECG after liraglutide injection on April 13.

**Figure 3:**
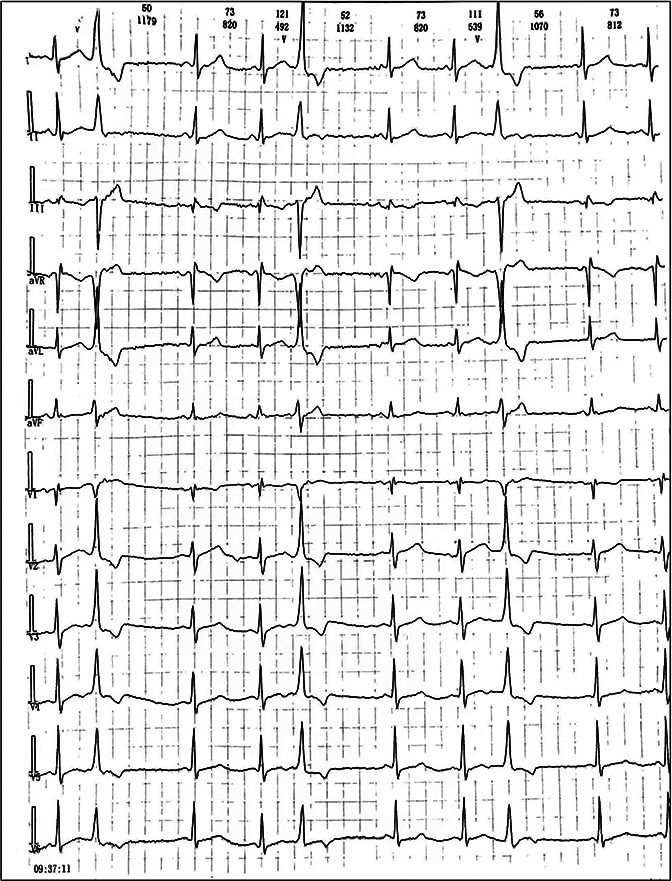
Ambulatory ECG after discontinuation of liraglutide on May 29.

We reported a case of increased premature ventricular contractions associated with liraglutide injection in China. Patient, male, 76 years old. He was admitted to the Department of Endocrinology on April 06, 2023, due to “10 years of elevated blood glucose and 4 months of poor control”. His original glucose-lowering regimen was oral acarbose tablets 50 mg tid, which was later changed to insulin glulisine (Sanofi Beijing Pharmaceutical Co., Ltd.) with a dose of 8 U in the morning, 10 U in the middle, and 4 U in the evening by subcutaneous injection and oral gliclazide tablets (Tianjin Huazin Pharmaceutical Co., Ltd.) 80 mg qd due to poor glycemic control. He had a 5-year history of coronary artery disease and underwent selective coronary angiography in our hospital on Feb. 10, 2023 reporting a mid LAD myocardial bridge with 90% stenosis in the middle and distal segments, flow TIMI grade 3, no significant stenosis in the LCX, RCA, flow TIMI grade 3, and implantation of 2.5*18 mm coronary rapamycin-targeted eluting stent in the anterior descending branch. The patient had no previous acute coronary syndrome or postoperative myocardial infarction, the patient's cardiovascular medications included sacubitril valsartan sodium tablets 100 mg qd (Beijing Novartis Pharmaceutical Co., Ltd.), bisoprolol fumarate tablets 1.25 mg qd (Merck KGaA, Germany), clopidogrel bisulfate tablets 75 mg qd (Hangzhou Sanofi Pharmaceutical Co., Ltd.), and atorvastatin calcium tablets 20 mg qn (Pfizer Pharmaceuticals Ltd.), and on Feb. 8, 2023, cardiac ultrasound showed widening of the ascending aorta (3.5 cm of the ascending aorta), enlargement of the left atrium (anterior-posterior left atrial diameter of 4.1 cm), left ventricular diastolic hypoplasia, cardiac arrhythmia, and an EF (%) of 53%. On February 09, the holter electrocardiogram showed: average heart rate: 62 beats/min, sinus rhythm, frequent premature ventricular beats, premature ventricular beats: 3,804 beats, occasional premature atrial beats, ST-T changes (Figure 1). Physical examination: BP: 138/76 mmHg, P: 70 beats/min, no abnormalities on cardiopulmonary auscultation, height: 172 cm, weight: 70 kg, BMI: 23.66 kg/m^2^. HbA1c: 7.40%, troponin, myoglobin, creatine kinase isoenzyme, FT3, FT4, TSH, hepatic and renal function, electrolytes, urinary albumin creatinine ratio were negative. Electromyography showed bilateral posterior tibial motor nerve conduction velocity slowed down. Resting electrocardiogram showed sinus rhythm and T-wave changes (Ⅰ, aVL, V1–V6). After admission, the patient was treated with insulin pump for hypoglycemic therapy, and the hypoglycemic regimen was adjusted to liraglutide (Novo Nordisk, Denmark) 0.6 mg qd, subcutaneous injection at 07:00 on April 10. At 14:00 on April 10, the patient began to develop obvious palpitation without obvious cause. When the symptoms occurred, BP was 120/60 mmHg and HR was 75 beats/min. Immediately bedside electrocardiogram showed frequent ventricular premature beats, and urgent tests of troponin, myoglobin, and creatine kinase isoenzyme were negative. On April 11, he reported no relief of palpitation. The resting electrocardiogram showed sinus rhythm, suspected ST segment elevation ≤0.05 mv in leads Ⅱ and aVF, frequent premature ventricular contractions, and negative troponin, myoglobin, and creatine kinase isoenzyme. On April 13, the patient's electrocardiogram showed: average heart rate: 79 beats/min, sinus rhythm, occasional atrial premature beats, frequent ventricular premature beats, ventricular premature beats: 10,229 beats, ST-T alteration, heart rate variability analysis: heart rate variability indices SDNN (50), SDANN (47), RMSSD (10), PNN50 (0) decreased (Figure 2). On April 14, liraglutide was stopped and insulin glulisine was given subcutaneously 6 U in the morning, 4 U in the evening and 4 U before meals. The symptoms of palpitation were relieved. One week after discharge, the patient was injected with liraglutide 0.6 mg qd again due to the large fluctuation of blood glucose. After the use of liraglutide, the patient again developed obvious palpitation. On April 23, liraglutide was stopped and instead use insulin glulisine subcutaneously, and the palpitation was relieved after discontinuation of liraglutide. The patient did not continue to use liraglutide for one month and had no palpitation symptoms. On May 29, the reexamination of holter showed: average heart rate: 59 beats/min, sinus rhythm, episodic atrial premature, episodic ventricular premature, ventricular premature beats: 860 beats, T-wave alterations, heart rate variability analysis: heart rate variability indexs SDNN (94), SDANN (89), PNN50 (0) decreased (Figure 3).

## 3 Discussion and conclusion

Glucagon-like peptide-1 receptor agonists (GLP-1RAs) are a class of drugs used for the treatment of type 2 diabetes and obesity. Liraglutide is the world's first long-acting human GLP-1 analogue used in the treatment of type 2 diabetes mellitus (T2DM). In addition to its hypoglycemic effect, it can also contribute to weight, lower blood pressure, regulate lipids, and comprehensively and effectively intervene in a variety of cardiovascular risk factors (4). The common adverse reactions to liraglutide are gastrointestinal discomfort, including nausea, vomiting, diarrhea, and a few patients will develop pancreatitis, tachycardia, etc. According to the 2023 ADA Standards for the Diagnosis and Treatment of Diabetes, it is recommended that GLP-1RAs or SGLT2i be added to patients with type 2 diabetes who are at high risk for atherosclerotic cardiovascular disease or cardiovascular risk, regardless of whether their glycated hemoglobin is within the standard range, as long as there are no contraindications (5).

**Figure 4:**
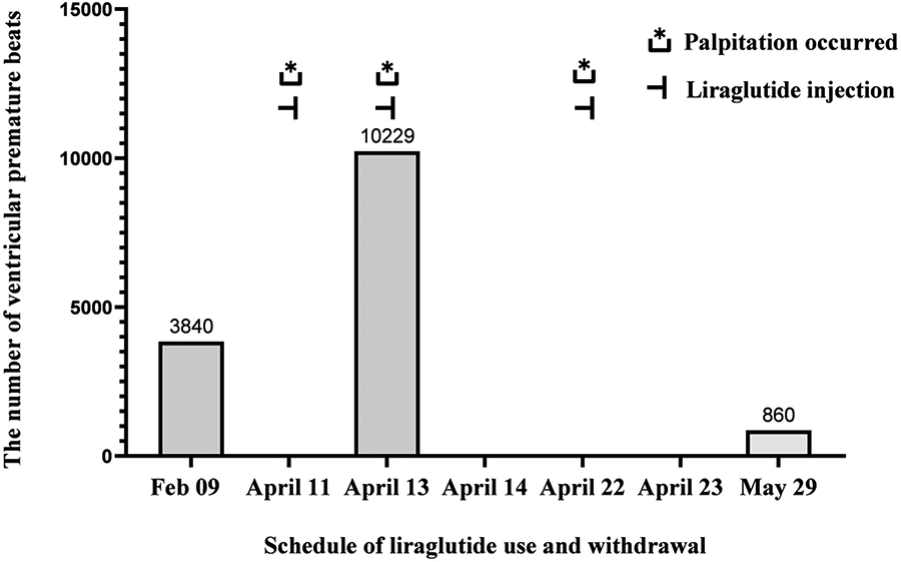
Schedule of liraglutide use and withdrawal.

In this case report, a patient with type 2 diabetes and cardiovascular disease was treated with liraglutide following the ADA criteria for the diabetes management, but subsequently developed significant arrhythmias. We speculate that this may be related to liraglutide, as there was a significant temporal correlation between the history of liraglutide injections and ventricular arrhythmias, as well as the palpitation symptoms. However, the patient was unable to undergo the holter on April 22 (Figure 4). The symptoms of palpitations caused by organic lesions such as hyperthyroidism, pheochromocytoma, electrolyte disturbance, fever, anemia, and psychological factors can be effectively ruled out by considering the medical history, resting electrocardiogram, dynamic electrocardiogram, and laboratory tests. Saudek CD et al. (6) compared the effects of implantable insulin pump (IIP) therapy vs. multiple daily insulin (MDI) injections on cardiovascular risk factors in patients with type 2 diabetes requiring insulin therapy. The study found that IIP therapy reduced weight gain, hypoglycemia, and other associated adverse effects. Derosa G et al. (7) similarly compared the effectiveness of IIP therapy with MDI therapy, and found that continuous subcutaneous insulin infusion (CSII) treatment seems to reduce the rates of cardiovascular events (CV) compared with MDI therapy. Moreover, CSII also improved glycemic control without increasing the number of hypoglycemia. According to the results of ambulatory glucose monitoring (Table 1), the Time In Range (TIR) gradually increased, and the Time Below Range (TBR) remained at 0% after the injection of liraglutide, which could rule out palpitations caused by hypoglycemia, and for the time being, excludes arrhythmia due to the insulin pump.

**Table 1:** Ambulatory glucose monitoring.

Core indicators 2023-04-06—2023-04-12 (7 days)										
Dates	Number of measurements	Time in range (TIR)	Percentage of time <3 mmol/L	Percentage of time below target range	Percentage of time above target range	Percentage of time >13.9 mmol/L	Average glucose	Standard deviation (SD)	Mean amplitude of glycemic excursions (MAGE)	Coefficient of variation (CV)
04–12	653	90.7%	0%	0%	9.3%	0%	7.5	1.8	5	24%
04–11	1,440	91.58%	0%	0%	8.42%	0%	7.78	1.7	3.97	21.85%
04–10	1,440	84.21%	0%	0%	11.58%	4.21%	7.61	2.5	5.57	32.85%
04–09	1,440	42.11%	0%	0%	49.47%	8.42%	10.18	2.6	5.96	25.54%
04–08	1,440	54.74%	0%	0%	34.74%	10.53%	9.64	3.1	6.38	32.16%
04–07	1,440	75.79%	0%	0%	9.47%	14.74%	8.94	3.8	8.17	42.51%
04–06	653	54.55%	0%	0%	22.73%	22.73%	10.31	3.7	4.97	35.89%

GLP-1 receptor agonists may reduce cardiovascular risk through direct and indirect effects on common cardiovascular risk factors such as blood pressure reduction, anti-inflammatory pathways, cardiac output, ischemic regulation, and endothelial function (8). We hypothesize that the increase in ventricular premature beats in our patient may be related to liraglutide injection, which has been the subject of fewer previous studies and reports. Additionally, the cause of the arrhythmia may be related to alterations in sympathovagal balance, including a drop in blood pressure in vasovagal syncope (9), and mimetic sympathetic activation after excess histamine production (10). Askin L et al. (11) reported that assessment of ambulatory blood pressure (ABP) and heart rate variability (HRV) in patients with premature ventricular contractions (PVCs) is part of a comprehensive approach to the assessment of cardiovascular regulation. Kumarathurai P et al. (12) observed the impact of liraglutide on HRV and diurnal variation of heart rate (HR) in overweight patients with newly diagnosed type 2 diabetes (T2D) and stable coronary artery disease (CAD), and this study found that overweight patients with CAD and newly diagnosed T2D, liraglutide increased HR and reduced HRV. Liraglutide induced changes in time and frequency domain parameters of HRV, which suggests an effect on sympathovagal balance. However, the relationship between liraglutide and increased ventricular premature beats is still unclear. GLP-1 can increase heart rate by enhancing sympathetic nervous system activation and reducing parasympathetic tone, or by direct involvement of GLP-1RA in the atrioventricular node (13). The meta-analysis by Wu S et al. (1) indicated that the overall arrhythmogenic risk of this class of drugs was low, and it was linked to obesity and high-dose usage. Lorenz M et al. (14) compared the extent and duration of the effects of different GLP-1RA on heart rate in healthy individuals and patients with T2DM. They found that long-acting GLP-1RA led to a significant and sustained increase in 24-h mean heart rate, while short-acting drugs caused a more temporary increase. However, studies have reported that an increase in heart rate, regardless of the magnitude, does not appear to elevate cardiovascular risk in patients with T2DM and those with (or at high risk for) cardiovascular disease. Jorsal A et al. (15) compared the effects of liraglutide with a placebo group on left ventricular systolic function in patients with stable chronic heart failure with or without diabetes mellitus. They found that liraglutide treatment was associated with an increase in heart rate and serious adverse cardiac events, including one case of ventricular tachycardia (VT), nonfatal VT, atrial fibrillation requiring intervention, exacerbation of ischemic heart disease, and one case of death due to worsening of heart failure. These events resembled the arrhythmias observed in our case report.

This case report suggests that clinicians should consider drug-related factors when using liraglutide in the treatment of diabetic patients with coronary heart disease and arrhythmia. Further clinical studies are needed to determine the precise relationship between liraglutide and the increased occurrence of ventricular premature beats. We hope that this clinical case report will attract the attention of endocrinologists and clinical pharmacists. Attention should be paid to whether liraglutide causes adverse reactions such as ventricular premature beats.

## Data Availability

The original contributions presented in the study are included in the article/Supplementary Material, further inquiries can be directed to the corresponding author.
